# Isolation of a Novel Metalloproteinase from Agkistrodon Venom and Its Antithrombotic Activity Analysis

**DOI:** 10.3390/ijms20174088

**Published:** 2019-08-21

**Authors:** Jin Huang, Hui Fan, Xiaojian Yin, Fang Huang

**Affiliations:** 1 State Key Laboratory of Natural Medicines, China Pharmaceutical University, Nanjing 210009, China; 2 Chinese Medicine College, China Pharmaceutical University, Nanjing 210009, China

**Keywords:** Agkistrodon venom, metalloproteinase, fibrinogen, antithrombotic, metabolomics

## Abstract

Snake venom contains large amounts of active proteins and peptides. In this study, a novel snake protein, metalloproteinase SP, was successfully isolated from the venom of *Agkistrodon acutus* by multi-gel chromatography. The isolated protein exhibits anti-platelet aggregation activity. Animal experiments showed that it exhibited defibration, anticoagulation, and antithrombotic effects and contributes to improved blood rheology and antiplatelet aggregation. In vivo experiments demonstrated that it prolonged clotting time, partial thromboplastin time, prothrombin time, thrombin time, fibrinogen time and reduced fibrinogen content of mice. Also, metalloproteinase SP inhibited carrageenan-induced tail thrombosis, ADP-induced acute pulmonary embolism, and ADP, Arachidonic acid (AA), or collagen-induced platelet aggregation. In vitro experiments showed that the protein cleaved the α, β, and γ chains of fibrinogen. Metabolomic analysis upon metalloproteinase SP treatment revealed that 14 metabolites, which are mainly involved in phenylalanine, tyrosine, and tryptophan biosynthesis, responded to metalloproteinase SP treatment. In summary, the isolated snake venom protein inhibits formation of acute pulmonary embolism probably through regulating and restoring perturbed energy, lipid, and amino acid metabolism.

## 1. Introduction

Thrombotic disease is one with a high morbidity and mortality rate in the world, accounting for approximately 40% of deaths yearly [1]. Thrombotic diseases include pulmonary embolism (PE), deep vein thrombosis (DVT), myocardial infarction, coronary atherosclerosis, ischemic stroke, and so on [2,3]. The formation of thrombus is closely related to platelet aggregation, blood coagulation, and fibrin network formation. At present, antithrombotic drugs are categorized into three groups: anti-platelet aggregation drugs, anticoagulants, and thrombolytic drugs.

Evidence from recent research indicates that snake venom contains many active protein or peptides with defibration, anticoagulation, antiplatelet aggregation, and antithrombotic functions [4]. The anti-thrombotic action of snake venom protein is due to its ability to cleave the fibrinogen, reduce the content of fibrinogen, activate fibrinolytic enzyme, inhibit the activation of FXa, FIIa, thrombin, and other coagulation factors [5,6,7]. A large number of snake venom protease components have been isolated and some snake venom preparations are widely used in clinical practice.

Deinagkistrodon is one of the most virulent snake species in China that mostly lives in China’s coastal and southwestern regions [8,9]. Snake venom contains a variety of active ingredients related to blood coagulation, such as phospholipase A2, serine protease, thrombin, metalloproteinase, and thrombin-like protein. Several new proteases and peptides have been isolated from the venom of Agkistrodon, such as Agacutas, Pt-A (Glu-Asn-Trp), Pt-B (Glu-Gln-Trp), ACH-11 [10,11,12]. These proteases affect many coagulation factors and cascades in the hemostatic system, such as cleavage of the alpha or beta chain of fibrinogen, inhibition of FXa, and activation of plasmin [13]. Most of the studied venomous snake venom polypeptides belong to serine proteases rather than metalloproteinases [14]. Metalloproteinases constitute an important components of the Agkistrodon genus. Metalloproteinase is an essential anticoagulant factor and exhibits fibrinogenolytic activity. However, there are few studies on the isolation and characterization of metalloproteinases from the venom of snakes belonging to the Agkistrodon genus. There is also paucity of information on the pharmacological effects of the isolated metalloproteinases. Metabolomics is an emerging discipline that compares the changes in body metabolites under different physiological and pathological conditions. In recent years, metabolomics has been widely used in drug research. It can be applied to study the mechanism of drug action by examining the changes of metabolic components in the body using serum, urine, and tissue fluid. It has been widely used in the study of monomeric compounds and traditional Chinese medicinal compounds. This study, to our best knowledge, is the first to report the anti-thrombotic effect mechanism of snake venom monomer using metabolomics

This work aimed to isolate and purify a novel metalloproteinase SP, determine its chemical properties and amino acid sequence, and investigate its pharmacological effects via in vivo studies. In order to study the mechanism of action of the novel snake venom protein, we investigated its antithrombotic effect using fibrinogen solubilization assay combined with UPLC-Q/TOF-MS-based non-targeted plasma metabolomics.

## 2. Results

### 2.1. Isolation of Metalloproteinase SP from Agkistrodon Venom

**Figure 1:**
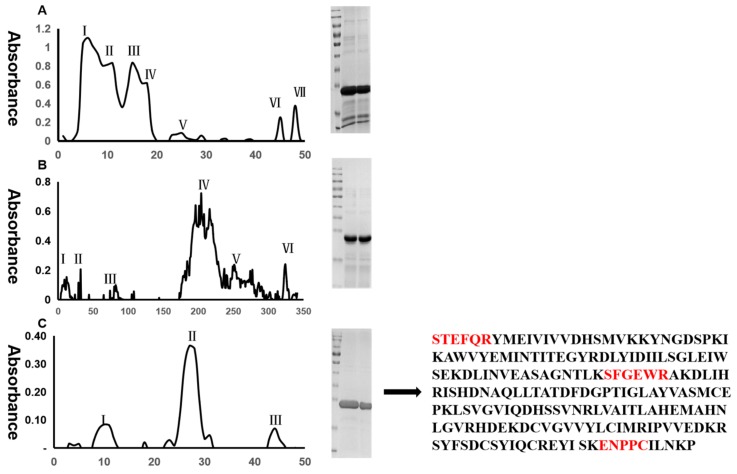
Isolation of metalloproteinase SP from Agkistrodon venom. (**A**) Snake venom (1000 mg) was subjected to Sephadex TM G-75 chromatography (1 × 100 cm) by eluting with 5 mM Tris-HCl (pH 7.4). The fraction III contains the target molecular weight protein. (**B**) Fraction III was further subjected to separation on DEAE-SphadexA-50 column (2 × 150 cm), eluted with 5 mM Tris-HCl (pH 7.4) and a segmented concentration gradient of 0.2 M NaCl. The fraction I contain the target molecular weight protein. (**C**) Fraction I was then separated on Sephadex TM G-75 column (1 × 50 cm), with 5 mM Tris-HCl (pH 7.4). Fraction II of this separation contains the target molecular weight protein. M, marker of protein molecular weight. (**D**) The peptide sequence of targeted protein. Red colors indicated unique peptides identified by LC-MS/MS, matching to targeted protein.

Based on the molecular weight differences of the snake venom proteins, an efficient separation and purification was achieved by the functional group ion exchange. The metalloproteinase SP snake venom monomer was then isolated and purified by sequential preparation, including ultracentrifugation, molecular exclusion chromatography on Sephadex TM G-75 (Figure 1A), anion exchange chromatography on DEAE-SphadexA-50 (Figure 1B), Sephadex TM G-75 (Figure 1C), and ultrafiltration.

### 2.2. Protein Identification

Based on LC-MS/MS analysis and database search analysis, it was found that three specific amino acid sequence fragments including SFGEWR, STEFQR, ENPPCILNKP were identified to belong to metalloproteinase SP (Figure 1D and Figure S1). Metalloproteinase SP consists of 202 amino acids with a molecular weight of 22.945 KDa and an isoelectric point of 5.78.

### 2.3. Anti-Thrombotic Activity

#### 2.3.1. Anti-Coagulant Activity

**Figure 2:**
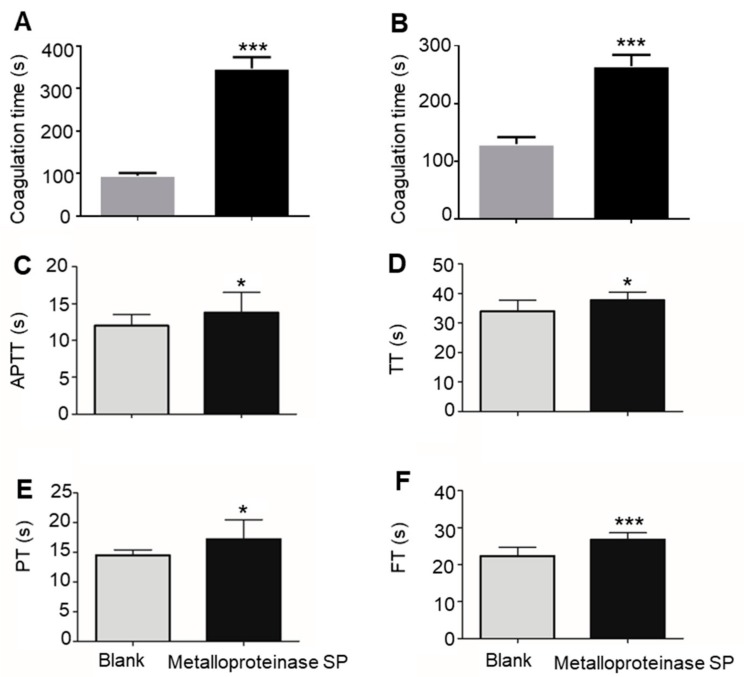
Anticoagulant activity of metalloproteinase SP was evaluated by coagulation time. (**A**) Blood coagulation time was determined by using the capillary technique. (**B**) Blood coagulation time was determined by the slide method. (**C**) Activated partial thromboplastin time (APTT). (**D**) Thrombin time (TT). (**E**) Prothrombin time (PT). (**F**) Fibrinogen time (FT). All data were expressed as mean ± SD, *n* = 10, * *p* < 0.05 and *** *p* < 0.01, compared with the blank group.

Compared to the control group, the coagulation time after metalloproteinase SP treatment was significantly prolonged (*** *p* < 0.001, Figure 2A,B). Anticoagulant activity of metalloproteinase SP was evaluated by the classical coagulation assays. The parameters including activated partial thromboplastin time (APTT), thrombin time (TT), prothrombin time (PT), and Fibrinogen time (FT) were checked (Figure 2C–F). Metalloproteinase SP protein also significantly prolonged the clotting time, which inevitably influenced the changes in blood components and affect blood rheology (Table 1 and Table 2).

#### 2.3.2. Anti-Thrombotic Activity

**Figure 3:**
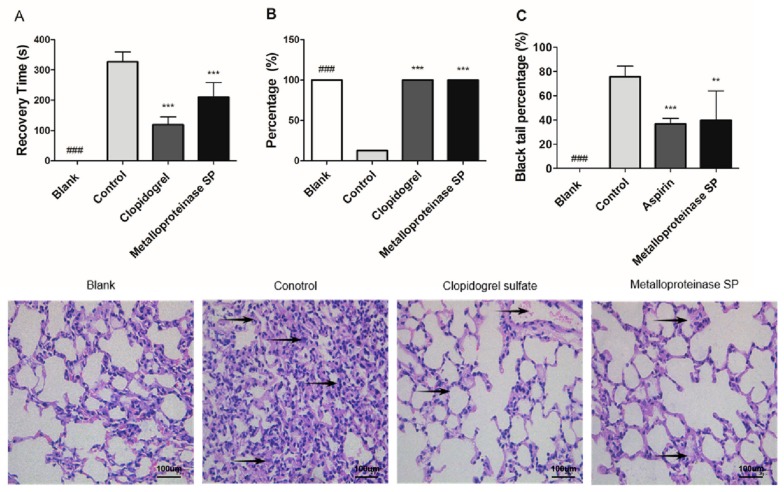
Effects of metalloproteinase SP on thrombosis. (**A**) Metalloproteinase SP inhibited the tail thrombus induced by the Carrageenan. (**B**,**C**) Metalloproteinase SP inhibited ADP-induced acute pulmonary thrombosis in mice. ADP (250 mg/kg) was intravenously injected to induce acute pulmonary thrombosis. The time that the mice righting reflex recovery was recorded as the recovery time. The hematoxylin and eosin stained section of the lung tissue shows the one for the control mouse to be dominated by thrombi. Effect of metalloproteinase SP on the ADP-induced formation of acute pulmonary thromboembolism in mice. Compared with the control group, the thrombus of the metalloproteinase SP and the clopidogrel group exhibited significant difference. All data were expressed as mean ± SD, compared with the control group, ** *p* < 0.01, *** *p* < 0.001 and compared with the blank group ^###^ *p* < 0.001.

**Figure 4:**
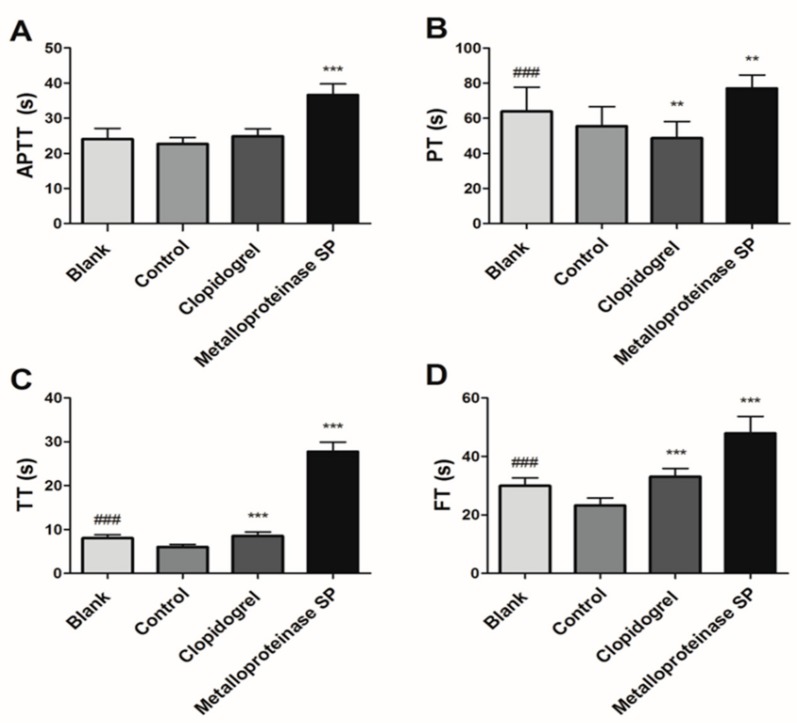
Effects of metalloproteinase SP on acute pulmonary embolism coagulation. Compared with blank group, the activated partial thromboplastin time (APTT), thrombin time (TT), prothrombin time (PT), and fibrinogen time (FT) of the control group exhibited significant difference (*** *p* < 0.001). Compared with control group, the metalloproteinase SP group could prolong the APTT, PT, TT, FT. (**A**) Activated partial thromboplastin time (APTT). (**B**) Thrombin time (TT). (**C**) Prothrombin time (PT). (**D**) Fibrinogen time (FT). All data were expressed as mean ± SD, compared with the control group, ** *p* < 0.01 ***, *p* < 0.001, and compared with the blank group ^###^ *p* < 0.01.

Compared with the blank group, the red blood cell and platelet content in the model group were significantly changed (** *p* < 0.01), indicating the successful establishment of model (Table 3). Metalloproteinase SP significantly inhibited acute pulmonary embolism formation induced by ADP (Figure 3A,B). It also inhibited Carrageenan-induced tail thrombus (Figure 3C and Figure S2). CCK-8 assay indicated that metalloproteinase SP did not have cytotoxicity within concentration of 0.5 ug/mL (Figure S3). Then, metalloproteinase SP activity was evaluated by the classical coagulation assays, thus, activated partial thromboplastin time (APTT), thrombin time (TT), prothrombin time (PT), and fibrinogen time (FT) (Figure 4A–D).

#### 2.3.3. Antiplatelet Aggregation Activity

**Figure 5:**
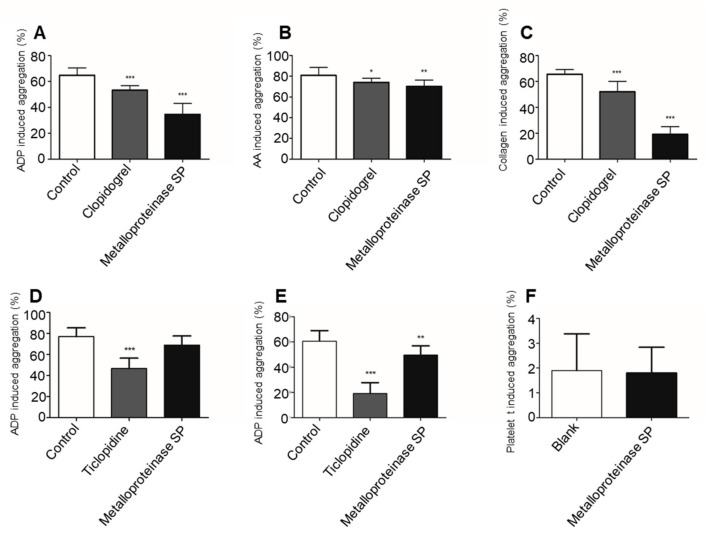
The in vitro and in vivo anti-platelet aggregation activity of metalloproteinase SP. (**A**) Compared with the blank group, the clopidogrel and the metalloproteinase SP groups specifically inhibited ADP-induced platelet aggregation. Metalloproteinase SP inhibited ADP-induced platelet aggregation with an inhibition rate of 44.28%. (**B**) Metalloproteinase SP inhibited AA-induced platelet aggregation with an inhibition rate of 13.19%. (**C**) Metalloproteinase SP inhibited collagen-induced platelet aggregation with an inhibition rate of 71.09%. The anti-platelet aggregation activity of metalloproteinase SP in vitro. (**D**,**E**) Metalloproteinase SP exhibited a dose-dependent inhibition of the ADP-induced aggregation of PRP. Compared with blank group, 0.5 μg of metalloproteinase SP significantly inhibited ADP-induced platelet aggregation with an inhibition rate of 18.27%. (**F**) Metalloproteinase SP not exhibited the promoted platelet aggregation activity. All data were expressed as mean ± SD, compared with the blank control group, * *p* < 0.05, ** *p* < 0.01, *** *p* < 0.01.

In vitro, metalloproteinase SP showed powerful antiplatelet activity by inhibiting the stimulatory effect of ADP, AA, and collagen (Figure 5A–C). In vivo, it exhibited a dose-dependent inhibition of the ADP-induced aggregation of PRP (Figure 5D–F). However, the inhibitory anti-platelet aggregation activity in vitro was more pronounced than in vivo.

### 2.4. Fibrinogenolytic Activity

**Figure 6:**
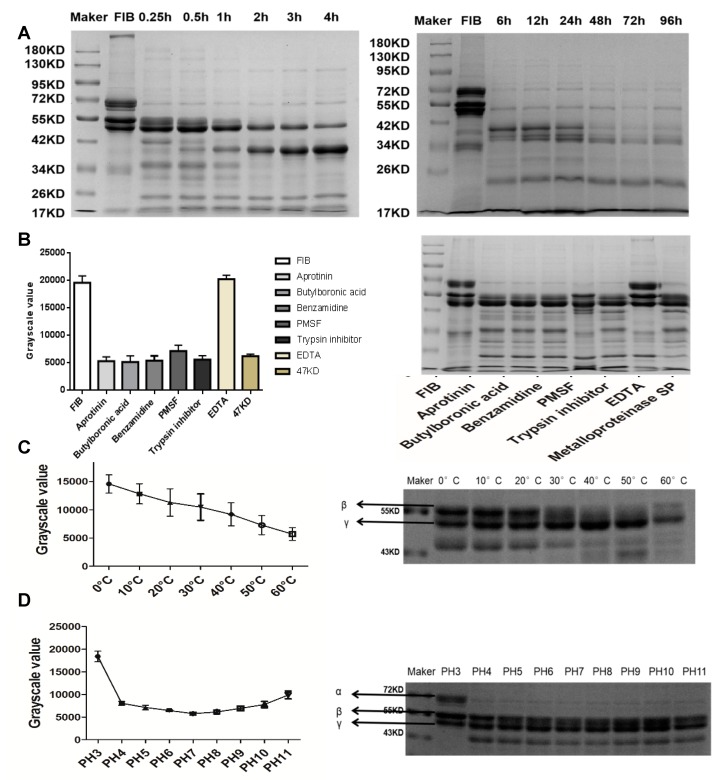
(**A**) Time-dependent effect of metalloproteinase SP on fibrinogen. Fibrinogenolytic activity was evaluated on 12% SDS-PAGE after incubation of metalloproteinase SP (5 μg) with human fibrinogen at 37 °C for different time periods. (**B**) Effects of inhibitors on metalloproteinase SP. Metalloproteinase SP was pre-incubated at 37 °C for 30 min at the presence of benzamidine (5 mM), ethylenediaminetetraacetic acid (EDTA, 5 mM), butyl boronic acid (20 mM), Trasylol (400 IU), trypsin inhibitor (20 mM), and PMSF (5 mM), respectively, prior to the addition of fibrinogen. (**C**) Temperature dependent effect of metalloproteinase SP on fibrinogen. Effect of temperature on fibrinogenolytic activity was evaluated on 12% SDS-PAGE after incubation of metalloproteinase SP (5 μg) with human fibrinogen at different temperature for 30 min. (**D**) The pH-dependent effect of metalloproteinase SP on fibrinogen. Effect of pH on fibrinogenolytic activity was evaluated on 12% SDS-PAGE after incubation of metalloproteinase SP (5 μg) with human fibrinogen at different pH for 30 min. All data were expressed as mean ± SD.

In vivo, metalloproteinase SP protein cleaved α, β, and γ chains of fibrinogen, and decomposed the fibrinogen chain sequence in the order, α > β > γ. In addition to degrading the α, β, and γ chains of fibrinogen, metalloproteinase SP also degraded the primary and secondary decomposition products to produce stable small molecular peptides (Figure 6A). Fibrinogen was also used to evaluate the effects of different inhibitors on the activity of metalloproteinase SP. The specific serine protease inhibitors such as benzamidine, butyl boronic acid, trasylol, trypsin, and PMSF were not able to significantly inhibit its enzymatic activity. However, EDTA significantly inhibited its enzymatic activity to a statistically significant level (Figure 6B). Regarding ideal temperature conditions, the enzyme showed high activity at 30–60 °C (Figure 6C). Metalloproteinase SP showed higher enzymatic activity at neutral pH values, with optimum catalysis at pH 6–7 (Figure 6D).

### 2.5. Effects of Snake Venom Monomer on the Chemical Constituents of Acute Pulmonary Embolism

**Figure 7:**
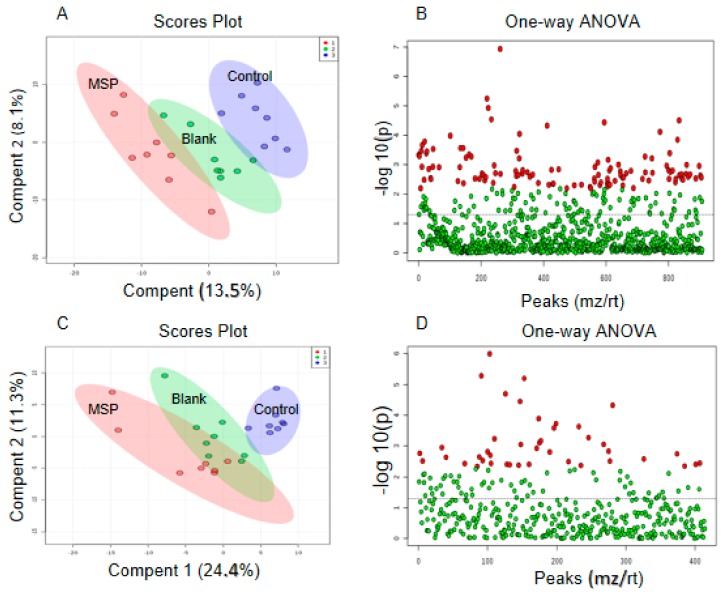
Score plot of plasma metabolomic profiling of three groups mice in positive ion mode (**A**) and negative ion mode (**C**). A total of 119 differential ions were screened by “adjusted *p*-value < 0.05, Fold change > 2” in positive ion mode (**B**). A total of 42 differential ions were screened by “adjusted *p*-value < 0.05, FC > 2” in negative ion mode (**D**).

In this study, the effect of metalloproteinase SP in an acute pulmonary embolism animal model was explored by analyzing metabolomic changes among metalloproteinase SP, model, and blank groups. As shown in the OPLS-DA score plot in both positive and negative ion modes, the three groups could be clearly distinguished (Figure 7A,C). This suggests that metalloproteinase SP exhibited a significant effect on acute pulmonary embolism by changing the anomalous metabolic states. Therefore, the metalloproteinase SP may be able to improve the pathological process of acute pulmonary embolism. The metabolites identified herein are listed in Table 4.

**Figure 8:**
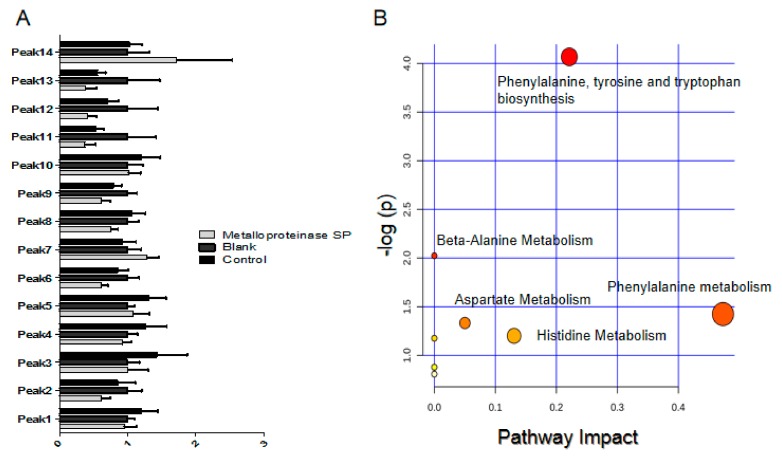
(**A**) Relative signal intensities for metabolic biomarkers in the blood. (**B**) Summary of pathway analysis with Metabo Analyst 4.0. Each ordinate in the plot represents a metabolic pathway. The ordinate is the original *p*-value obtained from the pathway analysis, and the abscissa is the influence value of pathways obtained from the topological analysis. The ordinate color and size of each circle was based on *p*-value and pathway impact value, respectively.

The potential metabolic pathways and networks were analyzed by Metabo Analyst 4.0. Based on the Met PA analysis of Metabo Analyst 4.0, the related biomarkers primarily participated in the following pathways: tyrosine and tryptophan biosynthesis, phenylalanine metabolism, catecholamine biosynthesis, beta-alanine metabolism, aspartate metabolism, histidine metabolism, propanoate metabolism, pyrimidine metabolism, valine, leucine and isoleucine degradation, and tyrosine metabolism (Figure 8, Table 5).

## 3. Discussion

The snake venom contains a large number of active proteins, peptides and small molecular weight compounds, which show anticoagulant, procoagulant, antithrombotic, antiplatelet aggregation, Fibrinogenolytic activity, analgesic, and other activities [15,16,17,18]. In the past few decades, various snake venom proteins have been extensively studied, and many snake venom proteins have been purified and characterized. Some snake venom preparations have been widely used in clinical settings as hemostatics, anticoagulants, antithrombotics, and thrombolytics. Examples include Ancrod, Batroxobin, and Acutase [18,19].

In the present study, metalloproteinase SP was purified form *Agkistrodon acutus* venom through ultracentrifugation, molecular exclusion chromatography on Sephadex TM G-75, anion exchange chromatography on DEAE-SphadexA-50, Sephadex TM G-75, and ultrafiltration. The molecular weight of metalloproteinase SP was determined by LC-MS/MS to be 22.9 kDa [20]. Metalloproteinase SP was proven to show anticoagulant, antithrombotic, antiplatelet aggregation, and fibrinogenolytic activities, and improve blood rheology. In vivo and in vitro experiments showed that metalloproteinase SP could cleave α, β, and γ chains of fibrinogen and further reduce fibrinogen content.

Fibrinogen plays a vital role blood coagulation, blood rheology, platelet aggregation, and thrombosis [21,22]. The coagulation process is traditionally classified into extrinsic, intrinsic, and common coagulation. In the common pathway of coagulation, fibrinogen forms the insoluble fibrin network under the stimulation of coagulation factors such as thrombin and FXa, which ultimately constitutes a fibrin clot that stops bleeding [23,24]. Fibrinogen mainly affects platelet aggregation, blood rheology, fibrinogen clot structure, atherosclerosis, plasmin activation, and fibrinogen decomposition (FgDP) to adjust the formation of thrombus [24,25,26,27,28]. Hence, the centrality of fibrinogen in platelet aggregation cannot be overemphasized. Fibrinogen has multiple GPIIb/IIa receptor binding sequences, such as γ400~411, α95~97, and α572~574 [29]. Fibrinogen functions as a bridge of platelet aggregation [29,30,31]. Moreover, fibrinogen is the most important factor affecting plasma viscosity. Increased plasma fibrinogen levels inevitably result in elevated plasma viscosity and shear stress, as well as endothelial cells and platelets activation. Activation of endothelial cells further promotes the expression or activation of various adhesion molecules and integrins, which leads to red blood cells adhesion and platelets aggregation and thrombosis [32,33,34]. Fibrin also possesses multiple plasminogen binding sites. Fibrinogen was cleaved by metalloproteinase SP to form fibrin, which may be through decomposing the α, β, and γ chain of fibrinogen, to reduce the content of the fibrinogen in the blood. Furthermore, it affected the blood coagulation pathway, prolonged the clotting time, directly inhibited the aggregation of platelets, indirectly regulated blood rheology, and indirectly regulated the formation of thrombus [35,36,37,38].

Based on the in vivo and in vitro experiments, the underlying mechanism of metalloproteinase SP on ADP-induced acute pulmonary embolism in mice was further investigated by untargeted mass spectrometry-based metabolomics profiling [39]. Metalloproteinase SP inhibited the formation of acute pulmonary embolism induced by ADP, which may be related to the amino acid metabolism and synthesis, including phenylalanine, tyrosine and tryptophan biosynthesis, phenylalanine metabolism, catecholamine biosynthesis, and eight other metabolic pathways [40,41].

Phenylalanine is one of the essential amino acids in humans that forms tyrosine under the action of phenylalanine hydroxylase in the liver [42,43]. Tyrosine synthesizes certain hormones and neurotransmitters such as dopamine (DA), norepinephrine (NE), epinephrine (E), and melanin in the nervous system and adrenal medulla. Thus, tyrosine plays an important role in regulating energy metabolism and scavenging free radicals [40]. Metalloproteinase SP regulated the biosynthesis of tyrosine and tryptophan, thereby alleviating lipid metabolism disorder and exert indirectly inhibited the formation of thrombus.

The content of catecholamines is closely related to blood coagulation and thrombosis [44]. Excessive levels of catecholamines could result in hypertension and acute myocardial infarction. Catecholamines act on platelet alpha (2)-adrenergic receptors to regulate platelet activation and aggregation, blood coagulation, and thrombosis.

The anticoagulant and antithrombotic activity of metalloproteinase SP may be ascribed to the regulation of histidine metabolism. Histidine is a semi-essential amino acid that is decarboxylated to form histamine under the action of histidine decarboxylase. Histamine exhibits diastolic vasoactive, antihypertensive and anti-inflammatory activities, and regulates a variety of allergic reactions. Histamine is clinically used for angina pectoris, cardiac insufficiency and other diseases [45]. Histamine has a strong vasodilator effect and is associated with a variety of allergic reactions and inflammation. Histidine may indirectly regulate inflammatory factors and inflammatory reaction by regulating the synthesis of histamine, thus inhibiting the formation of thrombus.

Metalloproteinase SP has many pharmacological activities, such as prolonging coagulation time, anti-platelet aggregation, inhibiting thrombosis, and decomposing fibrinogen. Metalloproteinase SP has high affinity with fibrinogen, and its anti-platelet aggregation and inhibition of thrombosis are closely related to its ability to decompose fibrinogen. Studies have shown that fibrinogen decomposition has an important relationship with the activation of fibrinolytic enzymes, and at the same time affects them. The experimental results showed that metalloproteinase SP can rapidly decompose fibrinogen and plays an anti-thrombotic role. However, fibrinogen content that is too low can easily lead to hemorrhage and coagulation disorders. At the same time, metalloproteinase has certain blood toxicity, which may lead to the reduction of platelet count and erythrocyte count. Thus, toxicity and safety of this venome component are needed to be further studied before its application in clinical. 

## 4. Materials and Methods 

### 4.1. Materials

SPF grade ICR mice (18–22 g) were purchased from the Comparative Medical Center of Yangzhou University (Jiangsu province, China, License No. SYXK (Su) 2018-0019). Sprague-Dawley rats (180–220 g) were purchased from Nanjing Qing long Shan Animal Center (Jiangsu province, China, License No. SYXK (su) 2018-0022). Animals were housed in a room of temperature of 22 ± 2 °C, relative humidity of 50 ± 5%, and exposed to 12 h dark/light cycle. The animals had free access to food and drinking water, and were allowed to acclimatize to their new environment for 1 week. The experiments were carried out in accordance with the guidelines and the regulations of the Ethical Committee of the China Pharmaceutical University. The protocols were approved by the Institutional Animal Care and Use Committee of the China Pharmaceutical University. The ethics approval number was AUC-37 (20180329) and the approval date was 29 March 2018.

### 4.2. Separation and Purification of Metalloproteinase SP 

A total of 1000 mg venom powder was dissolved in 5 mM Tris-HCl solution (pH 7.4), and then centrifuged at 12,000 r/min for 15 min at 4 °C. After centrifugation, the supernatant was collected and loaded on a gel filtration column of Sephadex TM G-75. The transparent supernatant was further analyzed by molecular exclusion chromatography with Sephadex TM G-75 column (1 × 100 cm, GE Healthcare, Frankfurt, Germany), which was pre-conditioned with 5 mM Tris-HCl (pH 7.4). Aliquots separated samples (4 mL/tube) were collected at a flow rate of 0.5 mL/min at 15 °C. The target component was chosen according to its profile on sodium dodecyl sulfate poly-acrylamide gel electrophoresis (SDS-PAGE) and was pooled, lyophilized, and then stored at −20 °C for subsequent chromatographic analysis. The target component from Sephadex TM G-75 was solubilized in 4 mL of 5 mM Tris-HCl (pH 7.4), and centrifuged at 12,000 r/min for 15 min at 4 °C. The supernatant was loaded on the anion exchange chromatography column (DEAE-SphadexA-50, 2 × 150 cm GE Healthcare, Germany) previously equilibrated and eluted with 5 mM Tris-HCl (pH 7.4). The target proteins were sequentially washed out with 5 mM Tris-HCl (pH 7.4), 0−0.2 M NaCl in 5 mM Tris-HCl (pH 7.4), and 0.2 M NaCl in 5 mM Tris-HCl (pH 7.4). The 4 mL/tube samples were collected at a flow rate of 0.5 mL/min at 15 °C. The target component was chosen based on its SDS-PAGE profile and was re-chromatographed after lyophilization. The clear supernatant was subjected to further purification via molecular exclusion chromatography using Sephadex TM G-75 (1 × 50 cm, GE Healthcare, Germany), previously equilibrated and eluted with 5 mM Tris-HCl (pH 7.4). Samples of 3 mL/tube were collected at a flow rate of 0.5 mL/min at 15 °C. The target component was chosen according to its profile on sodium dodecyl sulfate poly-acrylamide gel electrophoresis (SDS-PAGE) and was pooled, lyophilized, and stored at −20 °C for further chromatographic analysis.

### 4.3. Protein Quantification and Identification

The protein concentration was determined by the Bradford Protein Concentration Assay Kit (Thermo Fisher Scientific Incorporation, MA, USA), according to the manufacturer’s instructions [46]. The purified venome protein was separated by SDS-PAGE and identified by LC-MS/MS and a Uniprot database search (https://www.uniprot.org/uniprot/?query=agkistrodon+acutus&sort=score).

### 4.4. Pharmacological Evaluation of Metalloproteinase SP

#### 4.4.1. Anti-Coagulant Activity

##### Blood Clotting Assays

Half an hour after metalloproteinase SP intravenous administration via the tail vein, blood samples were collected from the orbital plexus of mice. Afterward, the time for the blood coagulate was recorded. The clotting time was observed when microfibril was visible. The average time of each observation was 20 s. The collected blood samples were also spotted on glass slides and clotting times determined. Clotting was achieved when the fibrin mesh appeared and could be picked by glass dissecting needle [47,48].

##### Blood Parameters Test

Half an hour after clopidogrel sulfate and metalloproteinase SP administration via the tail vein, blood samples were collected from the orbital plexus of mice or from abdominal aorta of mice. Both venous and arterial blood were anticoagulated by heparin sodium. The anticoagulant whole blood was placed in the automatic blood cell analyzer (RT-7600Vet, Rayto, Shenzhen, China) for detection and analysis within 2 h.

##### Coagulation Function Test 

One hour after the intravenous administration of test drugs or vehicles, blood samples were harvested from the orbital plexus. An aliquot of 600 μL blood was gently mixed with 66 μL citrate sodium anticoagulant. The changes of blood corpuscles in whole blood (anticoagulated by citrate sodium) were analyzed using blood cell analyzer RT-7600Vet (Rayto, Shenzhen China). After centrifugation at 3000 rpm/min for 15 min, the plasma and blood cells were collected and analyzed for four coagulation parameters using the semiautomatic blood coagulation analyzer LG-PABER-I (STEELLEX, Beijing, China) [49,50,51]. These parameters were: activated partial thromboplastin time (APTT), thrombin time (TT), prothrombin time (PT), and Fibrinogen time (FT). Standard analytical protocols were closely followed for all determinations.

#### 4.4.2. Anti-Thrombotic Activity

The anti-thrombotic effect of metalloproteinase SP was evaluated using two thrombosis pathological models [52,53].

##### ADP-Induced Acute Pulmonary Thrombosis in Mice

After metalloproteinase SP and clopidogrel treatment for 1 h, the ADP solution (250 mg/kg) was intravenously administered to induce acute pulmonary embolism, which could probably cause the paralysis or unpredicted death of mice. The recovery time of the mice was then recorded. After establishment of the model, the whole blood cell changes were assayed and coagulation parameters determined as earlier mentioned.

Half an hour after metalloproteinase SP intravenous administration via the tail vein, blood samples were collected from the orbital plexus of mice of mice. Both venous and arterial blood were anticoagulated by heparin sodium. The anticoagulant whole blood was placed in the automatic blood cell analyzer (RT-7600Vet, Rayto, Shenzhen, China) for detection and analysis within 2 h.

##### Carrageenan-Induced Tail Thrombosis in Mice

One hour after intravenous administration of metalloproteinase SP and aspirin in each group, Carrageenan solution (20 mg/kg) was intraperitoneally injected to induce tail thrombosis. The mice were then placed in environmentally-friendly cages for 12 h and their tail lengths recorded.

#### 4.4.3. Antiplatelet Aggregation Activity

##### In Vivo Antiplatelet Aggregation Assay

After 60 min of administration in each group, the rats were anaesthetized with 50 mg/kg chloral hydrate via abdominal injection. Blood samples were collected from abdominal aorta via cannulation. An aliquot of 8 mL blood was gently mixed with 880 μL citrate sodium anticoagulant (3.8%, *v/v*). Afterward, platelet-rich plasma (PRP) was obtained after centrifugation at 1000 rpm/min for 10 min. The residue was further centrifuged at 3000 rpm/min for 10 min to get platelet poor plasma (PPP). Prior to the antiplatelet aggregation assay, both PRP and PPP were incubated at 37 °C. An aliquot of 250 μL PPP was added into the measuring cup to calibrate the baseline. Thereafter, 225 μL of PRP was added and platelet aggregation stimulated with 25 μL different stimulants; thus, ADP (150 μM), AA (1 mg/mL), Collagen (50 μg/mL). Platelet aggregation was measured by aggregometer (AggRAM, Helena, USA), and the maximal aggregation rate was recorded within 5 min [54].

##### In Vitro Antiplatelet Aggregation Activity

The blank PRP plasma was harvested from control rats. Metalloproteinase SP (1 μg, 5 μg) was pre-incubated with 225 μL PRP at 37 °C for 5 min, and then stimulated with 25 μL different aggregating agents at the following final concentrations (ADP 15 μM). Platelet aggregation was assayed by a platelet aggregation instrument (AggRAM, Helena, TX, USA), and the maximum aggregation rate recorded in 5 min. 225 uL aliquot of PRP was measured into each cuvette and stimulated with metalloproteinase SP 25 uL (1 μg, 5 μg). Platelet aggregation was assayed by a platelet aggregation instrument, and the maximum aggregation rate recorded in 5 min [53].

#### 4.4.4. Fibrinogenolytic Activity

Human fibrinogen (8 mg/mL) was incubated with 5 μg metalloproteinase SP at 37 °C for 0.25 h, 0.5 h, 1 h, 2 h, 3 h, 4 h, 6 h, 12 h, 24 h, 48h, 72 h, and 96 h [53]. Afterward, the samples mixed with loading buffer solution were heated at 100 °C for 10 min. All samples were transferred to SDS-PAGE system for fibrinogen degradation analysis.

##### Effect of Temperature and pH on Metalloproteinase SP Activity

Human fibrinogen (8 mg/mL) was incubated with 5 μg metalloproteinase SP at 0 °C, 10 °C, 20 °C, 30 °C, 40 °C, 50 °C, and 60 °C for 30 min [55]. Additionally, 5 μg metalloproteinase SP was incubated with buffer solutions of different pH (pH 3, 4, 5, 6, 7, 8, 9, 10, 11) at 37 °C for 30 min, and subsequently incubated with human fibrinogen (8 mg/mL) at 37 °C for another 30 min. All samples were subjected to SDS-PAGE to investigate the effect of temperature and pH on metalloproteinase SP activity.

##### Effects of Serine Protease and Metalloproteinase Inhibitors on Metalloproteinase SP Activity

Different inhibitors, benzamidine (5 mM), ethylenediaminetetraacetic acid (EDTA, 5 mM), butyl boronic acid (20 mM), Trasylol (400 IU), trypsin inhibitor (20 mM), and PMSF (5 mM) were incubated with 5 μg metalloproteinase SP at 37 °C for 30 min [54]. The samples were further incubated with human fibrinogen at 37 °C for 30 min. All samples were analyzed by SDS-PAGE to evaluate the effect of inhibitors on metalloproteinase SP activity.

### 4.5. Effects of Snake Venom Monomer on the Chemical Constituents of Acute Pulmonary Embolism Based on UHPLC-Q/TOF-MS Non-Targeted Metabolomics

An aliquot of 150 μL of methanol/acetonitrile (3:1) solution (containing 0.4 μg/mL l-2-chloropheylaanine and 10 μg/mL ketoprofen as the internal standard for the ESI+ and ESI− modes respectively) was added to 50 μL plasma followed by vigorous vortex-mixing for 30 s. The mixture was centrifuged at 13,000 r/min at 4 °C for 10 min to precipitate protein. The supernatant (150 μL) was divided into two portions and dried under a gentle stream of nitrogen gas at room temperature. One of the samples was reconstituted with 75 μL of 50% aqueous acetonitrile for ultra-performance liquid chromatography with quadrupole time-of-flight mass spectrometry (UPLC-Q-TOF-MS) analysis in positive mode while the other was for negative mode. The quality control (QC) samples were prepared to improve the data quality for metabolic profiling.

#### 4.5.1. UHPLC/Q-TOF MS Analysis

Chromatographic evaluations were performed with an Agilent 1290 series (Agilent Corp, Santa Clara, CA, USA) HPLC system equipped with a binary pump, degasser, an autosampler, and a temperature-controlled column compartment. Chromatographic separations were achieved on an UPLC BEH C18 column (1.8 µm, 2.1 mm × 100 mm, Waters, Ireland) [25]. Mobile phase A was 0.1% formic acid aqueous solution and B was 0.1% formic acid-acetonitrile solution for the positive ion mode. Mobile phase A was 10 mM ammonium acetate solution and B was 10 mM ammonium acetate acetonitrile solution for the negative ion mode. This mobile phase system was run in a gradient elution program as follows: 90–99% A, at 0–1 min; 30–90% A, at 1–3 min; 15–30% A, at 3–8 min; 0–15% A, at 8–9 min; 0% A, at 9–10 min. The post-run time was 5 min. The oven temperature was set at 30 °C, the injection volume was 10 µL and the flow rate was 0.4 mL/min. 

Separated components were detected by Agilent 6545A Q/TOF mass spectrometer (Agilent Corp, Santa Clara, CA, USA) equipped with an ESI interface. The operating parameters were as follows: drying N_2_ gas flow rate, 8 L/min; temperature, 320 °C; nebulizer, 35 psig; capillary, 3000 V; skimmer, 65 V; OCT RFV, 750 V; fragmentor 100 V. The reference masses 254.2806 (ketoprofen) and 299.75 (L-Phenylalanine) were used for internal mass calibration during the runs in the positive and negative ion modes. At the scan rate of 1.50 spectra/s using fixed collision energies (0.00, 10.00, 20.00, 40.00 V) MS/MS data were acquired with isolation width MS/MS medium (~4 amu).

#### 4.5.2. Data Processing and Identification of Differential Metabolites

LC-MS raw data files were converted to m/z Data format using DA reprocessor (Agilent Corp., Santa Clara, CA, USA). Peak finding, filtering, and alignment were subsequently carried out using open-source R-Package XCMS [25]. Metabo Analyst 4.0 (https://www.metaboanalyst.ca/) was then employed for the analysis of the data. The use of Metabo Analyst 4.0 can be broadly categorized into three major activities: (1) missing value processing, (2) data filtering, and (3) data normalization (i.e., sample normalization, data transformation, and data scaling) [55]. 

Using Metabo Analyst 4.0 unsupervised principal component analysis (PCA), a general picture of the relationship that exists among the data matrix was obtained. Thereafter, the supervised orthogonal partial least-squares discrimination analysis (OPLS-DA) was carried out to examine the metabolite differences between the treatment, blank, and model groups. The inclusion criterion of the metabolites was that, the fold change between the groups compared should be greater than 1.2 and *p*-value < 0.05 (one-way ANOVA).

Metabolites that met this criterion were considered as the differential metabolites. In the identification of the biomarkers, the following databases were used: METLIN (http://metlin.scripps.edu/), HMDB database (http://www.hmdb.ca/), Metabo Analyst4.0 (https://www.metaboanalyst.ca/), SMPDB (http://www.smpdb.ca/) and KEGG (http://www.genome.jp/kegg/) [55,56] 

### 4.6. Statistical Analysis

Functional and enzymatic activities were assessed by two individual experiments in triplicate. All data were expressed as mean ± SD. The significance of differences was analyzed by one-way ANOVA followed by the Bonferroni correction. A value of *p* < 0.05 was considered statistically significant.

## 5. Conclusions

In this study, a novel metalloproteinase SP was isolated from Agkistrodon venom. In vivo, the metalloproteinase SP protein showed anticoagulant, antithrombotic, and antiplatelet aggregation activities and changed blood rheology. In vitro, metalloproteinase SP strongly inhibited platelet aggregation and cleaved the α, β, and γ chains of fibrinogen, but its activity was obviously affected by temperature or pH and could be reversed by EDTA. Finally, this study successfully built a mice model with acute pulmonary embolism to explore the antithrombotic mechanism of metalloproteinase SP. Assisted by LC-MS-Q-TOF-MS analysis, 14 representative biomarkers were identified, involving in phenylalanine, tyrosine and tryptophan biosynthesis, phenylalanine metabolism, catecholamine biosynthesis, and eight other metabolic pathways. All these findings indicated that metalloproteinase SP may affect blood coagulation and thrombus formation by decomposing fibrinogen and anti-platelet aggregation 

## Figures and Tables

**Table 1:** Hemodynamic examination of arterial blood in clopidogrel and metalloproteinase SP treated mice.

	Red Blood Cell Count (10^12^/L)	Hematocrit (%)	Blood Platelet Count (10^9^/L)	Plateletcrit (%)	Mean Platelet Volume (fL)	Platelet Volume Distribution Width (%)	Platelet Larger Cell Ratio (%)
Blank	5.50 ± 0.29	29.4 ± 1.81	812 ± 36.7	0.440 ± 0.03	5.45 ± 0.16	4.15 ± 0.20	5.93 ± 0.93
Clopidogrel sulfate	5.61 ± 0.22	29.2 ± 0.81	783 ± 27.5	0.430 ± 0.02	5.47 ± 0.41	4.22 ± 0.22	4.42 ± 0.50 ***
Metalloproteinase SP	5.47 ± 0.20	28.9 ± 0.96	777 ± 26.5 *	0.410 ± 0.02 **	5.42 ± 0.27	4.21 ± 0.30	6.45 ± 0.26

In the clopidogrel sulfate treated group, the platelet-larger cell ratio was decreased compared with the blank group. In the metalloproteinase SP group, the platelet count and plateletcrit were decreased compared to the blank group. All data were expressed as mean ± SD. * *p* < 0.05, ** *p* < 0.01, and *** *p* < 0.001 compared with the blank control.

**Table 2:** Hemodynamic examination of venous blood in clopidogrel and metalloproteinase SP treated mice.

	Red Blood Cell Count (10^12^/L)	Hematocrit (%)	Blood Platelet Count (10^9^/L)	Plateletcrit (%)	Mean Platelet Volume (fL)	Platelet Volume Distribution Width (%)	Platelet-Larger Cell Ratio (%)
Blank	6.20 ± 0.57	31.4 ± 2.51	701 ± 86.3	0.391 ± 0.04	5.46 ± 0.19	4.37 ± 0.20	6.21 ± 0.79
Clopidogrel sulfate	5.37 ± 0.31 **	27.9 ± 1.66 **	733 ± 99.5	0.382 ± 0.06	5.29 ± 0.21	4.45 ± 0.25	4.60 ± 0.94 **
Metalloproteinase SP	5.63 ± 0.24 *	30.1 ± 2.56	613 ± 61.6 *	0.341 ± 0.02 **	5.41 ± 0.29	4.36 ± 0.60	5.39 ± 1.21

In the clopidogrel sulfate treated group, red blood cell count, hematocrit, and platelet-larger ratio cell were decreased compared with the blank group. In the metalloproteinase SP treated group, the red blood cell count, the number of platelets and plateletcrit were decreased. All data were expressed as mean ± SD. * *p* < 0.05 and ** *p* < 0.01 compared with the blank group.

**Table 3:** Hemodynamic examination of venous blood to check clopidogrel and metalloproteinase SP effects on acute pulmonary embolism mice.

	Red Blood Cell Count (10^12^/L)	Hematocrit (%)	Blood Platelet Count (10^9^/L)	Plateletcrit (%)	Mean Platelet Volume (fL)	Platelet Volume Distribution Width (%)
Blank	8.01 ± 0.33	37.4 ± 2.36	865 ± 51.6	0.400 ± 0.04	4.16 ± 1.83	4.15 ± 0.21
Control	7.54 ± 0.40	34.6 ± 1.64	720 ± 73.5	0.350 ± 0.04	4.44 ± 0.65	3.99 ± 0.34
Clopidogrel sulfate	6.93 ± 0.16 **	31.3 ± 1.83 **	704 ± 56.9	0.320 ± 0.01	4.57 ± 0.167	3.91 ± 0.36
Metalloproteinase SP	6.92 ± 0.19 **	32.0 ± 1.16 **	640 ± 64.3 *	0.300 ± 0.03 *	4.70 ± 0.20	3.84 ± 0.28

In the clopidogrel sulfate treated group, red blood cell count and hematocrit were decreased compared with the blank group. In the metalloproteinase SP treated group, the red blood cell level, hematocrit, platelet count, and plateletcrit were decreased compared with blank. All data were expressed as mean ± SD. * *p* < 0.05 and ** *p* < 0.01 compared with the blank control group.

**Table 4:** The significantly changed metabolites in mice plasma after treatment with metalloproteinase SP.

Peak NO	Metabolite Name	Formula	Rt (min)	Molecular Weight	*p*-Value	FD	Control	Metalloproteinase SP	(+) MS	(+) MS/MS
1	5-Aminopentanoic acid	C5H11NO2	0.850	117.0790	*↓	0.7883	1.205 ± 0.241	0.952 ± 0.179	118.0861	118.086, 100.075, 72.080, 57.033, 56.049, 55.054, 44.049, 43.018
2	o-Tyrosine	C9H11NO3	1.100	181.0739	*↓	0.7218	0.857 ± 0.255	0.615 ± 0.131	182.0812	182.081, 165.05, 136.075, 119.040
3	Piperidine	C5H11N	1.467	85.0891	**↓	0.7029	1.431 ± 0.445	1.006 ± 0.295	86.0964	86.096, 69.070, 57.070, 56.050, 55.054, 52.033, 44.050, 43.054,
4	L-Isoleucine	C6H13NO2	1.483	131.0946	*↓	0.7330	1.261 ± 0.316	0.924 ± 0.138	132.1016	132.101, 86.096, 69.069, 44.049, 41.038
5	L-Phenylalanine	C9H11NO2	1.917	165.0790	*↓	0.8219	1.314 ± 0.249	1.083 ± 0.237	166.0856	166.086, 149.059, 131.049, 120.080, 107.049, 103.054
6	L-Norleucine	C6H13NO2	2.083	131.0946	**↓	0.7168	0.860 ± 0.153	0.616 ± 0.095	132.1009	132.10, 97.653, 86.695, 69.069, 44.048, 30.338
7	Palmitoyl Ethanolamide	C18H37NO2	3.767	299.2824	**↑	1.3987	0.917 ± 0.208	1.283 ± 0.183	300.2892	300.289, 282.279, 85.100, 83.085, 71.085, 67.054, 62.060, 57.069, 43.054,
8	beta-Alanine	C3H7NO2	0.617	89.0477	*↓	0.7330	1.071 ± 0.195	0.751 ± 0.110	88.0406	88.040, 71.013, 59.013, 53.003, 44.997, 43.018, 42.034, 41.002
9	L-Tyrosine	C9H11NO3	0.900	181.0739	*↓	0.7772	0.795 ± 0.116	0.618 ± 0.127	180.0664	180.066, 163.040, 119.050 72.009, 93.034, 74.024
10	Benzyl glycinate	C9H11NO2	1.916	165.0790	*↓	0.8400	1.208 ± 0.269	1.015 ± 0.174	164.0716	164.071, 91.055, 77.039
11	p-Cresol glucuronide	C13H16O7	2.267	284.0896	**↓	0.6891	0.540 ± 0.116	0.372 ± 0.158	283.0818	283.081, 265.073, 175.026, 117.019, 107.049, 87.008, 71.014, 43.018
12	p-Cresol sulfate	C7H8O4S	2.633	188.0143	**↓	0.5838	0.714 ± 0.159	0.417 ± 0.128	187.0073	187.007, 107.50, 105.334, 77.039
13	10-HDoHE	C22H32O3	4.800	344.2351	**↓	0.6892	0.559 ± 0.120	0.385 ± 0.164	343.2274	343.227, 325.210, 281.227, 189.130, 161.130, 153.092, 137.095, 109.102, 59.013
14	Octadec-9-enoic Acid	C18H34O2	10.150	282.4680	**↑	1.6569	1.033 ± 0.174	1.713 ± 0.826	281.2478	281.248, 263.238,59.0139

Compared to control group, “↑ and ↓” means the relative content of ions which is significantly increased or decreased. * *p* < 0.05, ** *p* < 0.01.

**Table 5:** Metabolic pathways and differential metabolites.

NO	Pathway	Hits	Metabolites
1	Phenylalanine, tyrosine and tryptophan biosynthesis	2	L-Phenylalanine, L-Tyrosine
2	Phenylalanine metabolism	2	L-Phenylalanine, L-Tyrosine
3	Beta-Alanine Metabolism	1	Beta-Alanine
4	Aspartate Metabolism	1	Beta-Alanine
5	Histidine Metabolism	1	Beta-Alanine

## Supplementary Materials

Supplementary Materials can be found at https://www.mdpi.com/1422-0067/20/17/4088/s1.

## Abbreviations

ANOVA	Analysis of variance
ADP	Adenosine diphosphate
AA	Arachidonic acid
RBC	Red blood cell
PLT	Platelet
PCT	Plateletcrit
HCT	Hematocrit
MPV	Mean platelet volume
PDW	Platelet volume distribution width
P-LCR	Platelet -larger cell ratio
