# Supplementary material for: Isolation of a Novel Metalloproteinase from Agkistrodon Venom and Its Antithrombotic Activity Analysis
Source: Int J Mol Sci. 2019 Aug 21;20(17):4088. doi: 10.3390/ijms20174088 (PMC6747553; doi:10.3390/ijms20174088)
Supplement: Supplementary file 1 [file ijms-20-04088-s001.pdf]

International Journal of
Molecular Sciences

MDPI

Supplementary material:

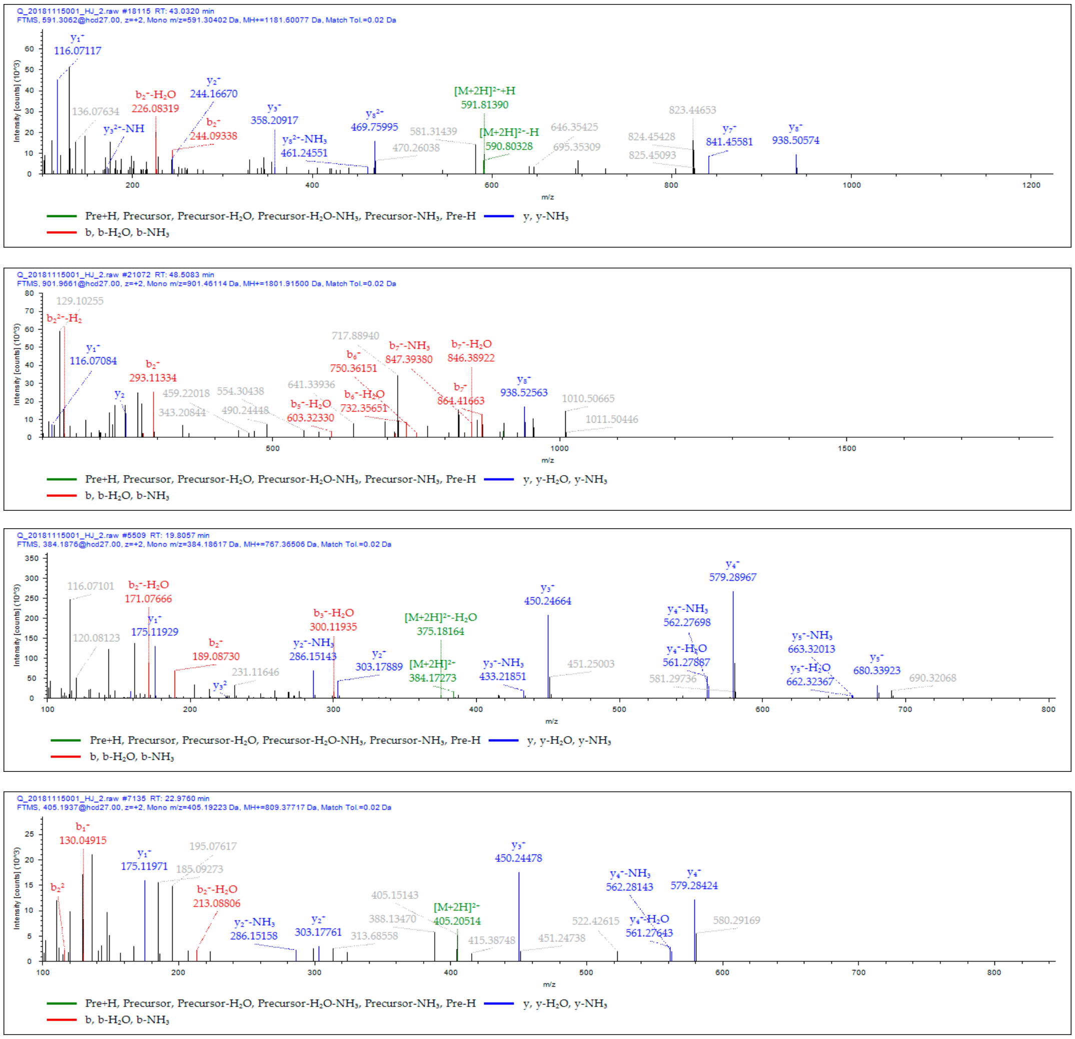

Figure S1. SP specific peptide map. Identification of snake venom protein monomer was based on LC-MS/MS and Uniprot (Universal Protein) database search.

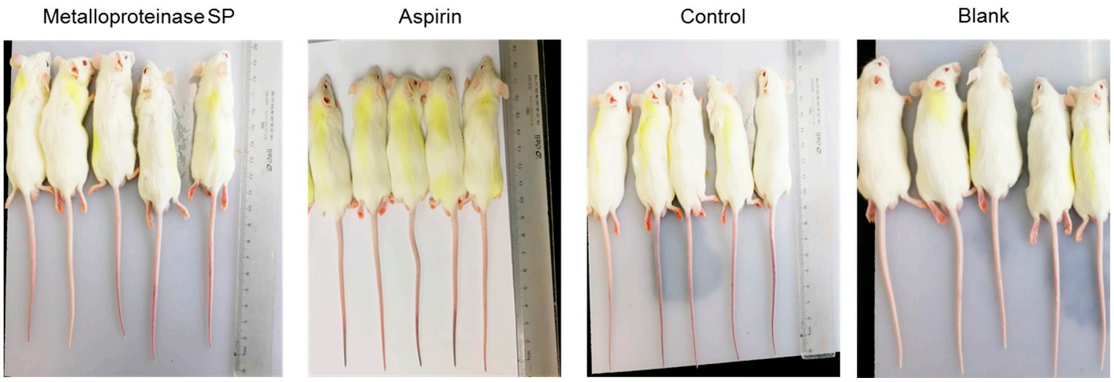

Figure S2. Carrageenan-induced tail thrombosis in mice. Metalloproteinase SP inhibited the tail thrombus by the Carrageenan which could decrease the tail thrombus length of the mice. Compared with the control group, the thrombus of the metalloproteinase SP and the clopidogrel group have significantly different.

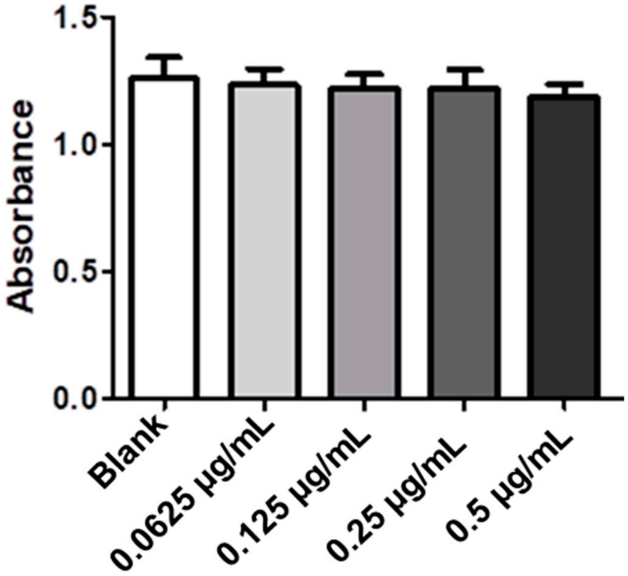

Figure S3. Cytotoxicity test of metalloproteinase SP. Cytotoxicity test was performed in HUVEC cells by CCK-8 assay. The absorbance value at 450 nm can indirectly indicate number of living cells. Within the dose of 0–0.5 ug/mL, there was no obvious toxicity and side effects of metalloproteinases.
